# Impact of Ag_2_O Content on the Optical and Spectroscopic Properties of Fluoro-Phosphate Glasses

**DOI:** 10.3390/ma12213516

**Published:** 2019-10-26

**Authors:** Marwa Ennouri, Luukas Kuusela, Ifa Jlassi, Bernard Gelloz, Laeticia Petit, Habib Elhouichet

**Affiliations:** 1 Laboratoire de Caractérisations, Applications et Modélisation des Matériaux LR18ES08, Sciences Faculty of Tunis, University of Tunis El Manar, Tunis 2092, Tunisia; ifa.jlassi@gmail.com; 2 Photonics Laboratory, Tampere University, FI-33101 Tampere, Finland; luukas.kuusela@tuni.fi (L.K.); laeticia.petit@tuni.fi (L.P.); 3 Graduate School of Engineering, Nagoya University, 2-24-16 Furo-cho, Chikusa-ku, Nagoya, Aichi 464-8603, Japan; gelloz@nuap.nagoya-u.ac.jp; 4 Physics Department, College of Sciences, University of Bisha, P.B. 551, Bisha 61922, Saudi Arabia or; 5 Sciences Faculty of Tunis, University of Tunis El Manar, Tunis 2092, Tunisia

**Keywords:** fluoro-phosphate glasses, Ag nanoclusters, erbium, energy transfer, optical gain

## Abstract

Glasses with the system (84.60-x) NaPO_3_-5 ZnO-(9.40-x) NaF-x Ag_2_O-1 Er_2_O_3_, (x = 0, 2, 4, and 6) (mol%) were synthesized by the conventional melt-quenching method. The impact of the addition of Ag_2_O on the physical, thermal, structural, and optical properties of the glasses is discussed. The Judd-Oflet analysis was used to evaluate the radiative properties of the emission transitions of the glasses. The enhancement of luminescence properties due to Ag_2_O is discussed in terms of consequent changes in the local electromagnetic field, symmetry, and the ligand field around the Er^3+^ ion. The heat treatment of the glass was performed in order to precipitate Ag nanoparticles (NPs), which form as a layer at the surface of the heat-treated glasses as confirmed using scanning electron microscopy (SEM). The Ag NPs were found to increase the intensity of the emission at 1.5 µm.

## 1. Introduction

Glass materials present several unique characteristics such as excellent transparency in wide spectral range, low cost, high optical damage threshold, and ease of doping with rare earth (RE) and transition metal ions [1]. Glasses have been of great interest for the development of various devices and optical components for photonics application due to their relatively easy manipulation. Glasses can be easily prepared in different shapes and sizes [2,3,4]. Among them, fluoro-phosphate-based glasses are considered as a prospective candidate due to their excellent transparency, low phonon energy, good mechanical and chemical stability, low melting point, and high ability to incorporate a large amount of rare earth dopants compared to silicate glasses, due to their two dimensional structure [5]. 

Recently, particular interest has been paid to glasses containing Ag nanoparticles (NPs) due to the spectacular improvement of luminescence properties, which benefit photonic applications [6,7]. In fact, collective charge density oscillations at the Ag NP surface, known as surface plasmon resonance (SPR), offers an opportunity to improve the photoluminescence (PL) properties of RE in glasses. In order to significantly enhance the PL, SPR absorption should overlap with the emission wavelength of RE. At present, the location of the absorption band relative to SPR depends on the size and morphology of the Ag NPs [8]. Approaching the particle size to the Fermi wavelength leads to discrete energy levels instead of a continuous band structure. The formed particle, in this case, is known as a nanocluster (NC) which is capable of intense light absorption and attractive tunable emission in the visible range [2]. Such characteristics are suitable for efficacy energy transfer (ET) from the NCs to RE ions, leading to a large enhancement of the luminescence in visible and near-infrared range [9]. 

The goal of this work is to improve the luminescence properties of Er^3+^ ions doped with fluoro-phosphate glass containing Ag species by seeking the best conditions to assure efficient energy transfer from silver NCs to Er^3+^ ions. Fluoride was reported to be suitable for the dispersion of the Ag NCs [10,11]. Furthermore, J–O analysis was performed to determine the radiative properties of Er^3+^ in the investigated glasses. The underlying mechanisms of luminescence intensity enhancement were clarified.

## 2. Materials and Methods

Sodium fluoro-phosphate glasses with compositions (84.60-x) NaPO_3_-5 ZnO-(9.40-x) NaF-x Ag_2_O-1 Er_2_O_3_ (in mol%) (x = 0, 2, 4, and 6, denoted as Ag0, Ag2, Ag4, and Ag6, respectively) were prepared by the conventional melt-quenching technique. The 10 g batches were melted in an electric furnace using quartz crucibles, in air atmosphere, at 875 °C for 5 min. The batches were prepared using (NaPO_3_)_6_ (Alfa Aesar, Tewksbury, MA, USA, 99.99%), NaF (Sigma Aldrich, Saint Louis, MO, USA, ≥99.0%), ZnO (Sigma Aldrich, Saint Louis, MO, USA, ≥99.5%), Ag_2_SO_4_ (Sigma Aldrich, Saint Louis, MO, USA, 99.999%), and Er_2_O_3_ (MV Laboratories Inc., Frenchtown, NJ, USA, 99.5%) as raw materials. The melt was then casted into a brass plate and annealed at 200 °C for 6 h, in order to avoid the internal mechanical stress. Afterward, the obtained samples were cut and optically polished for optical measurements. 

In order to analyze the composition and morphology of the investigated glasses, scanning electron microscopy (SEM)-type Zeiss Crossbeam 540 (Carl Zeiss, Oberkochen, Germany) equipped with Oxford Instruments X-MaxN 80 Energy Dispersive Spectroscopy Systems (EDS) detector (Oxford Instruments, Abingdon-on-Thames, UK) was used. The glasses were coated with a carbon layer. SEM was mainly used to characterize the cross section and the silver profile of the glasses. For this reason, samples were placed in the epoxy before the measurement. The accuracy of the elemental analysis was ±1.5 mol%.

The glass density was determined on bulk samples by a simple Archimedes method. Ethanol was used as the immersion liquid. The accuracy of the measurement was ± 0.02 g/cm^3^. 

Thermal properties of the glasses were measured using the Netszch F1 instrument (PerkinElmer Inc., Waltham, MA, USA) at a heating rate of 10 °C/min on glass powder. The measurements were performed on ~30 mg samples in an N_2_ atmosphere using platinum pans. All the temperatures were obtained with an accuracy of ±3 °C. The T_g_ (glass transition temperature) was determined as the inflection point of the endotherm which is the first derivative of the differential thermal analysis (DTA) curve. T_x_ and T_p_ were taken as the onset and the maximum point of the first exothermic peak, respectively.

The IR spectra of the glasses were obtained using Perkin Elmer Spectrum One FTIR Spectrophotometer (PerkinElmer Inc., Waltham, MA, USA) in attenuated total reflectance (ATR) mode in the range of 650–1500 cm^−1^. The spectra were normalized to the band with maximum intensity. 

Perkin Elmer lambda 1050 spectrophotometer (PerkinElmer Inc., Waltham, MA, USA) was used for the measurement of the UV–Vis near-infrared (NIR) absorption spectra. The absorption cross section σ_a_ (λ) was calculated using the following equation [12]:

(1) $$\sigma_{a}\left(\lambda \right)=\frac{\alpha_{a}\left(\lambda \right)}{N}$$

where $\alpha_{a}\left(\lambda \right)$ is the absorption coefficient at λ and N is the concentration of Er^3+^ ions (ions/cm^3^) calculated from the glass density. 

From the absorption spectra, the measured oscillator line strength (S_meas_) for each absorption transition was estimated according to the following equation [13,14]:

(2) $$S_{\mathrm{meas}}\left(J\to J\prime \right)=\frac{3ch\left(2J+1\right)}{8\pi^{3}{\lambda e}^{2}N_{0}}\left[\frac{9n}{{\left(n^{2}+2\right)}^{2}}\right]\int \alpha \left(\lambda \right)d\lambda$$

where, J and J’ are the total angular momentum quantum number of the initial and final states, respectively; c is the velocity of the light; h is the Planck constant; e is the charge of the electron; λ is the mean wavelength of the absorption band; and N is the concentration of Er^3+^ ions per unit volume. $\frac{9n}{{\left(n^{2}+2\right)}^{2}}$ represents the Lorentz local field correction for the ion in the dielectric medium and n is the refractive index. It was estimated using Equation (3) [9]:

(3) $$\frac{n^{2}-1}{n^{2}+1}=1-\sqrt{\frac{E_{g}}{20}}$$

where E_g_ is the optical band gap energy estimated by the Tauc model [15].

In addition, there was the theoretical oscillator line strength (S_cal_) introduced by Judd [13] and Ofelt [14]:

(4) $$S_{\mathrm{cal}}\left(J\to J\prime \right)=\underset{i=2,4,6}{\sum }\Omega_{i}{\left|\langle \left(S,L\right)J|\left|U^{i}\right||\left(S\prime ,L\prime \right)J\prime \rangle \right|}^{2}$$

where Ω_i_ (i = 1, 2, 3) are the Judd-Ofelt intensity parameters and $\left\|U^{\left(i\right)}\right\|$ are the doubly reduced matrix elements of the unit tensor operator $U^{\left(i\right)}$ of row i = 2, 4, and 6, which are evaluated from the ground state (|(S,L)J〉) to the upper state (|(S′,L′)J′〉) by the intermediate coupling approximation. Here, the reduced matrix elements values for Er^3+^ ion bands calculated by Carnall et al. were used [16]. An adjustment between the measured and the calculated oscillator strengths can be used to estimate the phenomenological Ω_2_, Ω_4_, and Ω_6_ parameters.

The quality of fit can be evaluated using the root-mean-square deviation determined by Equation (5) [4]:

(5) $$\Delta S_{\mathrm{rms}}={\left[{\left(q-p\right)}^{-1}\sum {\left(\Delta S\right)}^{2}\right]}^{1/2}$$

where q and p are the numbers of the used transition bands, and the unknown parameters (p = 3), respectively.

The spectroscopic quality factor (χ) can be determined by the equation [17]:

(6) $$\chi =\frac{\Omega_{4}}{\Omega_{6}},$$

The Judd-Ofelt intensity parameters were used to predict the radiative emission transition probabilities, A(J → J’) by the following equation [18]:

(7) $$A\left(J\to J\prime \right)=A_{\mathrm{ed}}+A_{\mathrm{md}}=\frac{64\pi^{4}e^{2}}{3h\left(2J+1\right)\lambda^{3}}\left[\frac{n{\left(n^{2}+2\right)}^{2}}{9}S_{\mathrm{ed}}+n^{3}S_{\mathrm{md}}\right]$$

where S_ed_ and S_md_ are the emission electric and magnetic dipolar line strengths, respectively. S_ed_ is determined by Equation (3) and presents a host medium dependence through the J–O parameters.

S_md_ is calculated by:

(8) $$S_{\mathrm{md}}=\frac{h^{2}}{16\pi^{2}m^{2}c^{2}}{\left|\langle \left(S,L\right)J|\left|L+2S\right||\left(S\prime ,L\prime \right)J\prime \rangle \right|}^{2}$$

where ${\left|\langle \left(S,L\right)J|\left|L+2S\right||\left(S\prime ,L\prime \right)J\prime \rangle \right|}^{2}$ are the reduced matrix elements of the operator L + 2S.

In our study, the values of A_md_ were deduced from Er^3+^-doped LaF_3_ crystal $A_{\mathrm{md}}^{\prime }$ values and corrected by considering the difference of the refractive index [19]:

(9) $$A_{\mathrm{md}}={\left(\frac{n}{n\prime }\right)}^{3}{A\prime }_{\mathrm{md}}$$

where n and n^’^ represent the refractive indexes of the investigated glass host and LaF_3_ crystal, respectively.

The radiative lifetime (τ_r_) of an emitting level can be determined from the spontaneous transition probability of the possible emission transitions from this level using the equation:

(10) $$\tau_{r}=\frac{1}{\sum_{J\prime }A\left(J\to J\prime \right)}$$

The fluorescence branching ratio β(J → J’) is related to the radiative decay rates by:

(11) $$\beta \left(J\to J\prime \right)=\frac{A\left(J\to J\prime \right)}{\sum_{J\prime }A\left(J\to J\prime \right)}=A\left(J\to J\prime \right)\tau_{r}$$

This ratio characterizes the possibility of attaining stimulated emission transition from any specific level.

The emission spectra in the visible were collected from bulk samples in air at room temperature using a multi-channel optical analyzer (spectrometer coupled to Charge Coupled Device (CCD) camera; Princeton Instruments, Acton, MA, USA) and a He-Cd laser (λ_exc_ = 325 nm). A filter (SCF-50S-38L, Sigma-Koki, Tokyo, Japan) was used to cut the excitation and record the PL signal above 390 nm. The IR emission measurements were recorded at room temperature with a 0.5 nm interval using a Jobin Yvon iHR320 spectrometer (Horiba Jobin Yvon SAS, Unterhaching, Germany) equipped with a Hamamatsu P4631-02 detector (Hamamatsu Photonics K.K., Hamamatsu, Japan) and a Thorlabs FEL 1500 filter (Thorlabs Inc., Newton, NJ, USA). The samples were excited with a single-mode monochromatic fiber (λ_exc_ = 976 nm) pigtailed laser diode (CM962UF76P-10R, Oclaro Inc., San Jose, CA, USA). In order to compare the emission intensities of samples, the emission spectra were collected from glasses crushed into powder and from bulk glasses with the same thickness. The errors on the measurement of the emission intensity were estimated to be less than 10%. 

The effective bandwidth (Δλ_eff_) was calculated from the emission band by the following equation [20]:

(12) $${\Delta \lambda }_{\mathrm{eff}}=\int \frac{I\left(\lambda \right)d\lambda }{I_{\max}}$$

where I(λ) is the emission intensity at λ wavelength and I_max_ is its maximum. The full-width at half-maximum (FWHM) can be also deduced from the emission bands.

The emission cross section (σ_e_) of the investigated glasses was obtained according to the Füchtbauer–Ladenburg method by [21]:

(13) $$\sigma_{e}=\frac{\lambda^{4}A_{R}}{8п{\mathrm{cn}}^{2}{\Delta \lambda }_{\mathrm{eff}}}$$

(14) $$A_{R}=\frac{8п{\mathrm{cn}}^{2}\left(2J\prime +1\right)}{\lambda_{p}^{4}\left(2J+1\right)}\int \mathrm{kdk}$$

where A_R_ is the spontaneous emission probability, ∫kdk is the integrated absorption cross section, ${\Delta \lambda }_{\mathrm{eff}}$ is the fluorescence effective linewidth, n is the refractive index, J’ and J are the total momentums for the excited and ground states, respectively, and λ_p_ is the absorption peak wavelength.

For the IR lifetime measurements, a photomultiplier tube (Hamamatsu Photonics H10330A-75, Shizuoka, Japan) coupled to an oscilloscope was used. The samples were excited using a laser diode (λ_exc_ = 980 nm). The measurements were performed from bulk glasses at room temperature. The errors of these measurements are estimated to be ±0.1 ms.

## 3. Results and Discussion

### 3.1. Thermal and Structural Properties of the Glasses

The thermal properties and the density of the glasses are displayed in Table 1.

As x increases, the glass transition and the crystallization temperatures decrease while the density increases. One notices that T_p_ increases when x increases from 2 to 6 suggesting that different crystals precipitate depending on Ag_2_O content. Furthermore, the increase of x slightly decreases the molar volume (V_m_) of the glasses. This indicates that the addition of Ag_2_O slightly decreases the interstitial spaces already presented in the glass network, as suspected from the decrease in the thermal properties. Therefore, the addition of Ag_2_O in the glass networks is suspected to result in a small reduction in the compactness and connectivity of the glass network [22]. Finally, the introduction of Ag_2_O into this glass matrix leads to a decrease in the thermal stability of the glasses as evidenced by the decrease in ΔT (ΔT = T_x_ – T_g_). Similar results were reported by Yuebo Hu et al. [23] for Tm^3+^/Er^3+^/Yb^3+^ co-doped oxyfluorogermanate glasses containing Ag nanoparticles.

**Figure 1:**
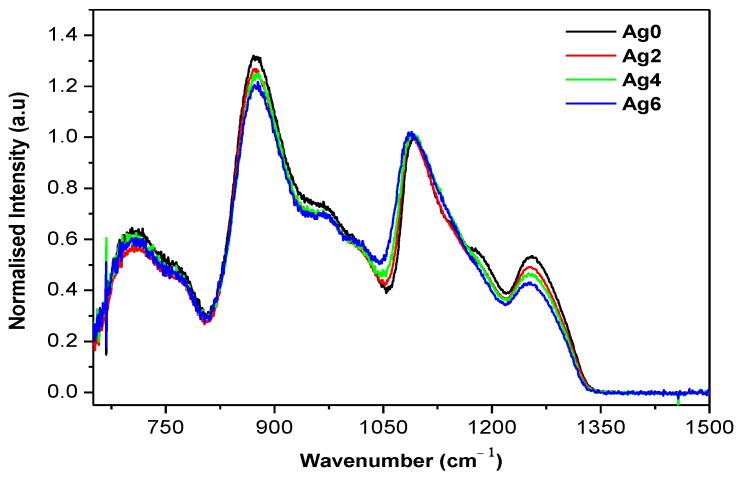
The IR absorption spectra of the investigated glasses.

The changes in the glass structure can be evidenced from the FTIR spectra presented in Figure 1.

The spectra exhibit four absorption bands located at ~1255, 1085, 875, and 700 cm^−1^ and also four shoulders at ~1183, 1015, 970, and 780 cm^−1^, which are all characteristics of the metaphosphate network. No IR band existed at the wavenumber range >1300 cm^−1^, where P = O vibration modes usually appear, which confirmed that these glasses were free of Q^3^ units. The attribution of the FTIR bands is listed in Table 2 [24,25,26,27,28,29].

As observed in Figure 1, an increase in Ag_2_O content decreases the number of Q^2^ units and increases the amount of Q^1^ units, as seen by the decrease in intensity of the bands at 875 and 1057 cm^−1^ compared to the intensity of the band at 1085 cm^−1^. Therefore, the addition of Ag_2_O is expected to depolymerize the glass network and form a greater number of non-bridging oxygens (NBOs). Similar results were reported by Mishra et al. [30] when adding Ag_2_O in glasses with the system 0.5P_2_O_5_-0.2CaO-0.2SrO-0.1Na_2_O. One also notices that an increase in Ag_2_O content shifts the IR band at 1092 cm^−1^ to lower wavenumbers suggesting that the addition of Ag_2_O decreases the covalence of the P-O-P [25,30]. This is in agreement with the decrease of T_g_ and V_m_ as x increases (Table 1).

### 3.2. Optical and Spectroscopic Properties of the As-Prepared Glasses

**Figure 2:**
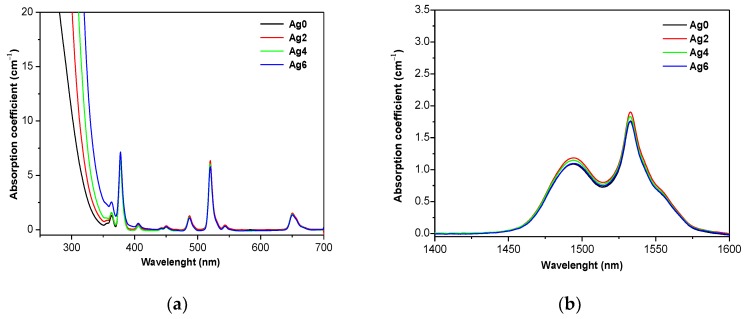
Absorption coefficient of the investigated glasses (**a**) in the UV–Vis and (**b**) IR range.

Figure 2 illustrates the UV–Vis–NIR absorption spectra of the investigated glasses.

The spectra exhibit absorption bands located at 1532, 980, 650, 543, 520, 487, 450, 405, and 376 nm which are due to the f–f transitions of the Er^3+^ ion, from the ground state ^4^I_15/2_ to their excited states ^4^I_13/2_, ^4^I_11/2_, ^4^I_9/2_, ^4^F_9/2_, ^4^S_3/2_, ^2^H_11/2_, ^4^F_7/2_, ^4^F_5/2_, ^4^F_3/2_, and ^2^G_9/2_, respectively. One notices that the optical band gap shifts progressively to a longer wavelength with the increase of x; this might be a consequence of the formation of a greater number of non-bridging oxygens (NBOs), as mentioned in the previous section. A similar shift in the band gap due to the addition of Ag_2_O was also reported in [30]. The presence of silver nanoparticles is known to lead to an absorption band at ~400 nm, due to the surface plasmon resonance (SPR) of Ag NPs [2,7]. As shown in Figure 2a, no absorption band at ~400 nm can be seen indicating that Ag are dispersed in the network in a small size of Ag species. As seen in Figure 2b, the addition of Ag_2_O has no significant impact on the shape of the absorption band centered at 1.5 µm, neither on the absorption cross sections at 980 nm and 1.5 µm (see Table 1) indicating that the Er^3+^ ions have similar sites in the investigated glasses. 

Judd-Ofelt theoretical analysis was investigated to predict important spectroscopic and laser parameters of the glasses. The J–O intensity parameters (Ω_t_), the root-mean-square deviation (ΔS_rms_) and the spectroscopic quality factor (χ) of our glasses compared with thus of other glass systems are tabulated in Table 3.

The low values of the adjustment quality (ΔS_rms_ < 0.2 10^−22^ cm^2^) is a clear indication of a good agreement between the experimental and the theoretical oscillator strengths S_mes_ and S_cal_. These results reflect the reliability of the fit. The Judd-Ofelt parameters of the investigated glasses are similar to the J–O parameters reported for Er^3+^-doped phosphate [31], fluoro-phosphate [32], and fluoride glasses [33]. Ω_2_ can be related to the covalency of a RE-ligand, the coordination symmetry of the environment around the Er^3+^ ion, and the polarizability of the ligand anions [6]. The slight increase in Ω_2_ when x increases confirms that the addition of Ag_2_O leads to minor changes in the Er^3+^ site: slightly less ionic Er^3+^-ligand bonds, lower symmetry around the Er^3+^ ions, and more polarized sites of the Er^3+^ according to [34]. The Ω_4_ and Ω_6_ parameters are related to the long-range effect of the glass host such as the rigidity, density, and other dielectric properties of the media [35,36]. The slight decrease of these parameters as x increases is probably associated with a decrease in the glass network rigidity, in agreement with structural analysis. 

The spectroscopic quality factor χ was found to slightly decrease from 0.78 to 0.65 with the increase of x indicating that the presence of Ag improves the fluorescence dynamics of the laser transition ^4^I_13/2_ → ^4^I_13/2_. One should point out that the spectroscopic quality factors of the investigated glasses are lower than those reported for phosphate glasses [1,32,33] but are included in the range of 0.126–3.372 reported for Er^3+^ in various glasses [37]. 

The values of the radiative transition probability (A), the fluorescence branching ratio β(J → J’), and the radiative lifetime (τ_r_) from the excited states ^4^I_13/2_, ^4^I_11/2_, and ^4^I_9/2_, to the ground state ^4^I_15/2_ of Er^3+^ in the investigated glasses are reported in Table 4.

The obtained values are in agreement with those reported in [38,39,40]. The branching ratio (β) values for the transitions ^4^I_13/2_ → ^4^I_15/2_, ^4^I_11/2_ → ^4^I_15/2_, and ^4^I_9/2_ → ^4^I_15/2_ were larger than 60%, confirming the possibility of achieving efficient IR and red emissions, under suitable excitation conditions [41]. For IR transitions, an increase in the radiative lifetime (τ_r_) accompanied by an increase of the radiative transition probability (A) was observed with an increase in x, confirming the improvement of emission dynamics by adding Ag_2_O.

**Figure 3:**
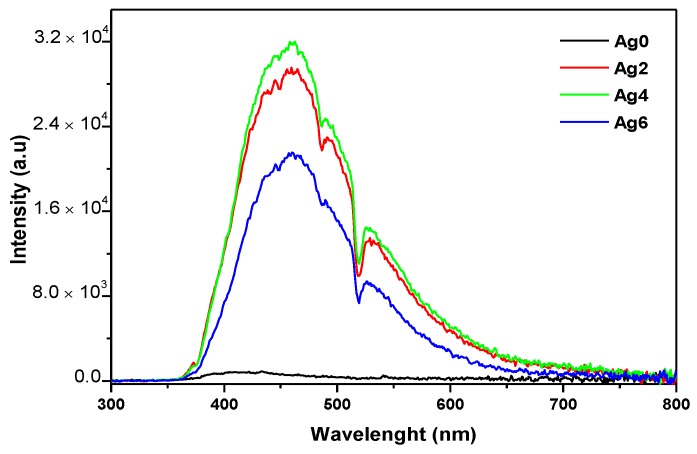
Emission spectra of the investigated glasses upon 325 nm excitation.

Figure 3 shows the emission spectra of the as-prepared glasses.

The spectra of the Ag-containing glasses exhibit a broadband covering almost the visible range. This band can be divided into three sub-bands located at blue, green, and red regions. The band in the blue region can be related to the emission of isolated Ag^+^ ions [42]. The green and red bands are due to the emission of molecule-like Ag nanoclusters (ML-Ag NCs) with different sizes [10]. The dominance of the blue emission indicates that the networks contain a large density of Ag^+^ and nanoclusters having a small size. One notices that an increase of x from 0 to 4 increases the intensity of the emission indicating an increase in the number of the Ag species. However, as x increases from 4 to 6, the intensity of the emission decreases. Due to the high Ag amount in the glass, the Ag–Ag distance is suspected to be reduced, thus increasing the interactions between the different Ag species. The spectra also show all the absorption bands relative to Er^3+^ ions, which appear in superposition of the broad emission band of Ag species; the higher the emission related to Ag species, the more absorption from Er^3+^ ions. Such overlapping suggests that energy transfer from excited Ag species to Er^3+^ ions might occur. Such a process was reported by Wei et al. [43] for oxyfluoride glasses containing Ag species and doped with Eu^3+^ ions. 

Note that a very weak luminescence from the Ag0 sample was detected which can be assigned to the intrinsic defects in the glassy host. In addition, the absence of Er^3+^ emission indicates that the 325 nm excitation is not a resonant excitation for Er^3+^ ions. 

**Figure 4:**
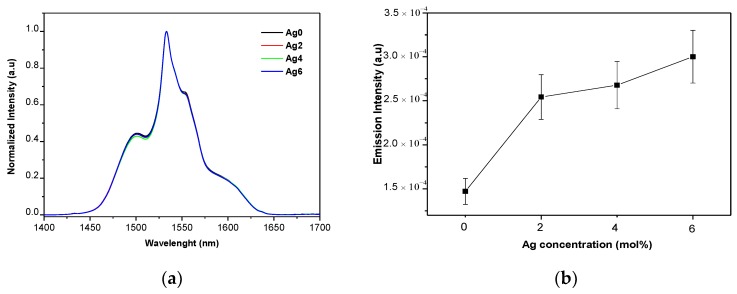
(**a**) Normalized emission band; (**b**) intensity of the emission at 1.53 µm as a function of x.

Figure 4a shows the normalized IR emission spectra of the investigated glasses.

The spectra exhibit a broad emission centered at ~1532 nm, due to the ^4^I_13/2_ → ^4^I_15/2_ transition of the Er^3+^ ion. This broad band is typical of the emission from Er^3+^ located in an amorphous host. One notices that the shape of the emission remains similar independently of x, confirming that Ag has no major impact on the Er^3+^ sites. An increase in x from 0 to 2 increases the intensity of the emission as seen in Figure 4b. However, all the Ag_2_O-containing glasses exhibit similar intensity of emission, independent of the concentration of Ag_2_O. 

The ^4^I_13/2_ lifetimes are listed in Table 5.

In agreement with changes in the intensity of the emission seen in Figure 4b, the lifetime increases when x increases from 0 to 2 and remains constant for any further increase in x. As the absorption cross section at 980 nm remains unchanged as x increases, the increase in the intensity of the emission at 1.5 µm can be related to the isolated Ag^+^ and the small molecule-like Ag (ML-Ag). 

Note that the absorption of a photon with a 980 nm wavelength followed by a second absorption in the excited state is equivalent to an absorption at 490 nm that coincides with the spectral position of the second PL band of Figure 3. Thus, even when the excitation wavelength is far from the stated density of the ML-Ag particle, it is possible that the excitation is in a resonant process from the nonlinear phenomenon [44]. 

Other laser properties of the glasses are listed in Table 6. 

An increase of x from 0 to 2 leads to an increase in the effective bandwidth (Δλ_eff_) probably induced by some modifications of ligand fields in the vicinity of Er^3+^ from one site to another [3,45]. However, as x increases from 2 to 6, Δλ_eff_ remains unchanged confirming that the Er^3+^ ions have a similar site in the Ag-containing glasses. The bandwidth quality factors, estimated from σ_e_* FWHM, are summarized in Table 6. The larger the product, the wider gain bandwidth and higher pumping efficiency. The bandwidth quality factors are similar to those reported for phosphate glass (179.73 10^−21^ cm^2^·nm) [46] and bismuth glass (280.60 10^−21^ cm^2^·nm) [47]. It is worth pointing out that the sample doped with 2 mol% Ag_2_O (Ag2) has a large bandwidth quality factor (205.82 10^−21^ cm^2^·nm) indicating that this glass may be a good candidate for an Erbium doped fiber amplifier (EDFA) host. 

### 3.3. Impact of Heat Treatment on the Optical and Spectroscopic Properties of the Glasses

**Figure 5:**
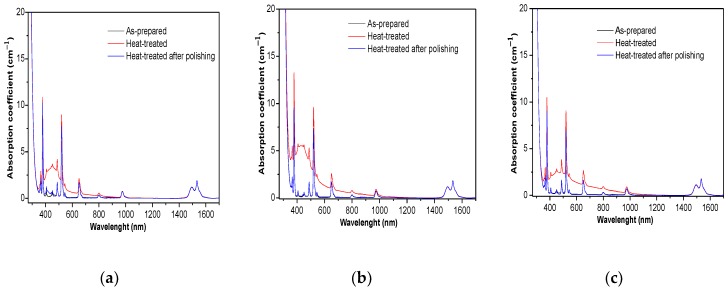
Absorption spectra of the heat-treated glasses: (**a**) Ag2, (**b**) Ag4, and (**c**) Ag6.

The glasses were heat-treated for 17 h at T_g_ + 10 °C in order to grow silver nanoparticles as performed in [6]. After heat treatment, a change in the color of the glasses from pink to yellow was seen. The absorption spectra of the heat-treated glasses are shown in Figure 5.

After heat treatment, a new broad band centered at ~412 nm appears in the absorption spectra. According to [48], this band can be related to the surface plasmon resonance (SPR) absorption of Ag NPs. During the heat treatment process, the viscosity of the glass decreases and therefore the ML-Ag species are suspected to coalesce to form Ag NPs. The asymmetric character of the SPR band indicates that the average distance between Ag particles may be much larger than their sizes as suggested by Malta et al. [49]. The intensity of this SPR band increases with the increase of x from 2 to 4 but decreases when x increases to 6. We think that the strong dipolar interactions between the isolated ML-Ag and Ag^+^ block their association, thus limiting the formation of Ag NPs. 

**Figure 6:**
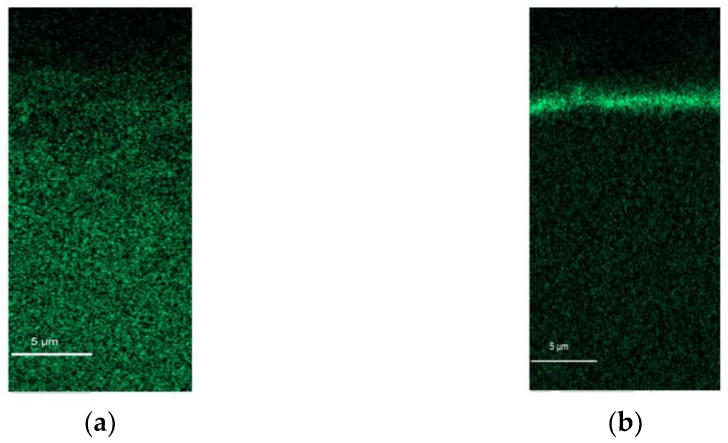
Elemental mapping of Ag in Ag2 (**a**) prior to and (**b**) and after heat treatment at the surface of samples.

After polishing of heat-treated glasses, the color of the glasses changed back to pink in agreement with the disappearance of the SPR absorption band (Figure 5). We suspect the Ag NPs to form only at the surface, as confirmed by the elemental mapping of Ag taken at the cross section of the glasses before and after heat treatment. As seen in Figure 6a, the elemental mapping of Ag clearly shows that Ag species are well dispersed in the glass.

After heat treatment (Figure 6b), the Ag species migrate to the surface and form a layer of Ag species on the surface of the glasses.

**Figure 7:**
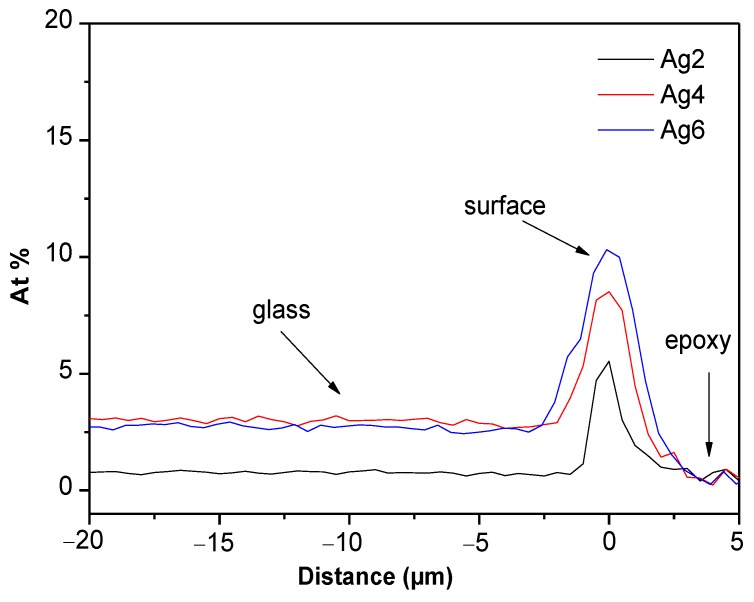
Ag concentration profile measured from the surface of the heat-treated glasses.

As seen in Figure 7, an increase in x increases not only the thickness of the Ag layer precipitating at the surface of the glass but also the Ag content in the layer. 

**Figure 8:**
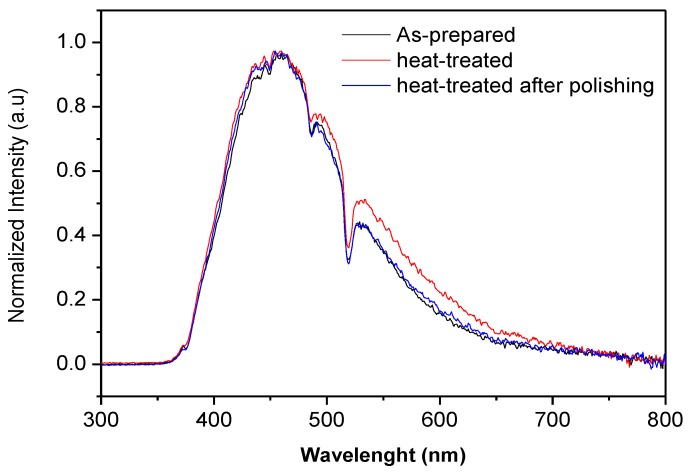
Normalized visible photoluminescence (PL) spectra of Ag_2_ glasses taken as an example.

The normalized visible emission spectra of the sample doped with 2 mol% of Ag_2_O (Ag2) prior to and after heat treatment are shown in Figure 8. 

The heat treatment had no noticeable effect on the intensity or on the shape of the emission except for the green band. The increase of the intensity of the shoulder at ~510 nm is a direct consequence of the Ag NCs growing to larger sizes during the heat treatment. It is noticeable that after polishing, the emission spectrum is similar to that of the as-prepared sample confirming that the newly formed Ag species are only at the surface. The same results were obtained for the other glasses.

**Figure 9:**
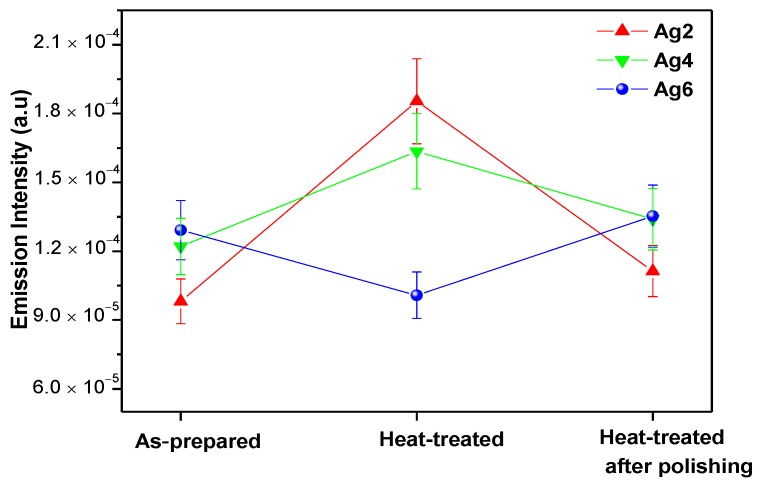
Variation of 1.53 μm band peak emission intensity.

The emission intensities of the heat-treated glasses prior to and after polishing are depicted in Figure 9.

Considering Er^3+^:^4^I_13/2_ lifetime values of these glasses (Table 6), the changes in the intensity of the emission and lifetime are similar: after heat treatment, they increase for the glasses doped with 2 (Ag2) and 4 mol% (Ag4) but decrease for the glass with 6 mol% (Ag6). The intensity of the emission at 1.53 µm and the lifetime increase after heat treatment probably due to the local field enhancement induced by the Ag NPs formed after heat treatment. A strong local electric field around Er^3+^ ions increases their transition probability from the ^4^I_13/2_ level to ^4^I_15/2_ level [50,51]. The decrease in the emission and in the lifetime of the glass with x = 6 (Ag6) after heat treatment is probably due to a reverse energy transfer from Er^3+^ ions to formed Ag species as suggested by [52]. After polishing, the intensity of the emission and the lifetime are back to the level of the emission from the glasses prior to heat treatment confirming that the newly formed Ag species are only at the surface. 

## 4. Conclusions

In summary, the addition of Ag_2_O in sodium fluoro-phosphate glasses contributes to the depolymerization of the glass network and creation of a greater number of terminal NBOs, as evidenced by the decrease in T_g_ and the red-shift of the optical band gap. However, there was no significant impact of Ag_2_O addition on the Er^3+^ sites to their absorption and emission cross sections. From the visible emission, the as-prepared glasses contain isolated Ag^+^ and molecular like (ML)-Ag NCs. The increase in the IR emission intensity and in the Er^3+^:^4^I_13/2_ lifetime were observed by adding 2 mol% of Ag_2_O probably due to the energy transfer from Ag species to Er^3+^ ions. However, the intensity of the emission did not increase when the concentration of Ag_2_O increased to 6 mol%. The glasses were heat-treated to precipitate Ag nanoparticles (NPs) as confirmed by the appearance of a new absorption band at ~400 nm and increase in the intensity of the emission at 1.5 µm and in the Er^3+^:^4^I_13/2_ lifetime. However, after polishing the heat-treated glasses, the glasses exhibited similar optical and luminescence properties to that of the as-prepared glasses indicating that the Ag NPs precipitated mainly at the surface of the glasses as confirmed by the elemental mapping of Ag. The enhancement in the spectroscopic properties of the glasses induced by the Ag species shows that the Er^3+^-doped and Ag NPs containing fluoro-phosphate glass are promising glass candidates for 1.53 μm band EDFA and for solid-state laser.

## Figures and Tables

**Table 1:** Thermal properties and density of the glasses.

Sample	T_g_ ± 3 °C	T_x_ ± 3 °C	T_p_ ± 3 °C	ΔT = T_x_ − T_g_ ± 6 °C	ρ (±0.02 g/cm^3)^	V_m_ (mol·cm^3^)
Ag0	297	390	457	95	2.67	36.75
Ag2	290	343	375	53	2.75	36.67
Ag4	285	345	384	60	2.84	36.46
Ag6	280	340	392	60	2.93	36.27

**Table 2:** FTIR bands attribution.

Wavenumber (cm^−1^)	Attribution	Reference
770	ν_s_(P-O-P) of Q^2^ units	[24]
875	ν_as_(P-O-P) of Q^2^ units in chain	[24]
960	ν_as_(P-O-P) of Q^2^ units in small rings	[25,26]
980	ν_s_(PO_3_^2−^) in Q^1^ units	[27]
1015	ν_s_(PO_3_F) bonds	[28]
1020	ν_as_(P-O-P) of Q^2^ units in large rings	[25,26]
1085	ν_as_(P-O-P) of Q^1^ units v_s_(P-O-P) of Q^2^ units	[29]
1183	ν_as_(P-O-P) in Q^2^ units	[27]
1255	ν_as_(O-P-O) of Q^2^ units	[25]

ν(P-O-P) are the phosphorus-oxygen-phosphorus fundamental vibrations.

**Table 3:** Judd-Ofelt parameters Ω_i_ (i = 2, 4, and 6), spectroscopic quality factor (χ), and root-mean-square deviation (ΔS_rms_) of Ag/Er^3+^-doped glasses.

Sample	Ω_2_ (10^−20^ cm^2^)	Ω_4_ (10^−20^ cm^2^)	Ω_6_ (10^−20^ cm^2^)	χ	ΔSrms (10^−22^)
Ag0	3.41	0.81	1.08	0.75	0.09
Ag2	3.50	0.72	1.02	0.70	0.08
Ag4	3.62	0.66	0.97	0.68	0.08
Ag6	3.65	0.60	0.91	0.65	0.19
Phosphate [31]	3.89	1.01	0.55	1.83	-
Fluoro-phosphate [32]	4.90	1.37	1.27	1.08	-
Fluoride [33]	2.91	1.27	1.11	1.14	-

**Table 4:** Calculated radiative parameters of Ag/Er^3+^ co-doped glasses.

Sample Code		Ag0	Ag2	Ag4	Ag6	ZnO-AlF_3_ [38]	PKAZFEr10 [39]	SAMEA0.9 [40]
^4^I_13/2_ → ^4^I_15/2_	A (s^−1^)	209.04	220.25	220.11	219.73	148	177.18	485
	β_JJ’_ (%)	1.00	1.00	1.00	1.00	1.00	1.00	1.00
	τ (ms)	2.09	2.20	2.20	2.19	6.79	5.64	2.61
^4^I_11/2_ → ^4^I_15/2_	A (s^−1^)	210.87	226.79	220.89	219.21	174.25	206.09	137
	β_JJ’_ (%)	0.83	0.84	0.83	0.83	0.89	0.86	0.84
	τ (ms)	2.51	2.69	2.63	2.61	5.08	4.16	6.14
^4^I_9/2_ → ^4^I_15/2_	A (s^−1^)	154.01	142.55	145.86	143.06	-	83.46	183
	β_JJ’_ (%)	0.66	0.63	0.64	0.64	-	0.53	0.89
	τ (ms)	2.30	2.24	2.23	2.25	-	6.40	4.83

**Table 5:** Er^3+^:I_13/2_ measured lifetime (τ_m_) values of the investigated glasses (±0.1 ms).

	Measured Lifetimes		
Sample	As-Prepared	Heat-Treated	Heat-Treated After Polishing
Ag0	1.3	-	-
Ag2	1.7	1.9	1.7
Ag4	1.7	1.8	1.6
Ag6	1.5	1.2	1.5

**Table 6:** Optical properties of as-prepared glasses.

Glass Sample	Er^3+^ (10^20^ ions cm^−3^) ±5%	α at 1.5 μm (cm^−1^)	Δλ_eff_ (nm)	σ_a_ at 1.5 μm (10^−21^ cm^2^) ±10%	σ_e_ at 1.5 μm (10^−21^ cm^2^) ±10%	FWHM (nm)	σ_e_*FWHM (10^−21^·nm)	G (P = 1) (cm^−1^)
Ag0	3.277	1.759	37	5.57	4.61	36	165.97	1.51
Ag2	3.284	1.902	57	5.97	5.02	41	205.82	1.66
Ag4	3.303	1.829	57	5.74	4.86	37	179.82	1.61
Ag6	3.320	1.750	56	5.46	4.69	37	173.53	1.56

FWHM: full-width at half-maximum.
